# Outcomes of ovarian transposition in gynaecological cancers; a systematic review and meta-analysis

**DOI:** 10.1186/1757-2215-7-69

**Published:** 2014-06-25

**Authors:** Kumar Gubbala, Alex Laios, Ioannis Gallos, Pubudu Pathiraja, Krishnayan Haldar, Thomas Ind

**Affiliations:** 1 Gynaecologic Oncology Unit, Churchill Hospital, Oxford University Hospitals NHS Trust, Oxford, UK; 2 Department of Gynaecologic Oncology, Royal Marsden Hospital NHS Trust, London, UK; 3 School of Clinical and Experimental Medicine, College of Medical and Dental Sciences, University of Birmingham, Birmingham, UK

## Abstract

**Background:**

Pelvic irradiation is essential for improving survival in women with pelvic malignancies despite inducing permanent ovarian damage. Ovarian transposition can be performed in premenopausal women in an attempt to preserve ovarian function. As uncertainty occurs over the proportion of women who are likely to benefit from the procedure, we performed a systematic review and meta-analysis of the proportion of women with ovarian function preservation, symptomatic or asymptomatic ovarian cysts and metastatic ovarian malignancy following ovarian transposition.

**Methods:**

Medline, Embase and The Cochrane Library databases were systematically searched for articles published from January 1980 to December 2013. We computed the summary proportions for ovarian function preservation, ovarian cyst formation and metastatic ovarian disease following ovarian transposition by random effects meta-analysis with meta-regression to explore for heterogeneity by type of radiotherapy.

**Results:**

Twenty four articles reporting on 892 women undergoing ovarian transposition were included. In the surgery alone group, the proportion of women with preserved ovarian function was 90% (95% CI 92–99), 87% (95% CI 79–97) of women did not develop ovarian cysts and 100% (95% CI 90–111) did not suffer metastases to the transposed ovaries. In the brachytherapy (BR) ± surgery group, the proportion of women with preserved ovarian function was 94% (95% CI 79–111), 84% (95% CI 70–101) of women did not develop ovarian cysts and 100% (95% CI 85–118) did not suffer metastases to the transposed ovaries. In the external beam radiotherapy (EBRT) + surgery ± BR group, the proportion of women with preserved ovarian function was 65% (95% CI 56–74), 95% (95% CI 85–106) of women did not develop ovarian cysts and 100% (95% CI 90–112) did not suffer metastases to the transposed ovaries. Subgroup meta-analysis revealed transposition to the subcutaneous tissue being associated with higher ovarian cyst formation rate compared to the “traditional” transposition.

**Conclusion:**

Ovarian transposition is associated with significant preservation of ovarian function and negligible risk for metastases to the transposed ovaries despite common incidence of ovarian cysts.

## Introduction

Ovarian transposition (OT) has proven invaluable for ovarian function preservation in patients with pelvic malignancies requiring pelvic irradiation. First described in 1958 [1], it was initially performed at laparotomy until more recently when laparoscopic and robotic techniques have been described [2,3]. Surgical approaches include intra- and retroperitoneal transposition on or lateral to the psoas muscle, the paracolic gutters, percutaneous needle transposition of the ovaries and exteriorisation to the subcutaneous fat tissue [4-8].

It has now been established as a simple and reliable method with reduced morbidity [9]. Standard criteria for the preservation and transposition of the ovaries have been proposed [10]. In effect, the procedure is limited to a population of young premenopausal patients with early-stage, operable cervical tumours with a need for primary or adjuvant radiotherapy. It can be additionally performed for ovarian dysgerminomas, vaginal cancers but also non-gynaecological malignancies like ependymomas, Hodgkin’s disease, sarcomas and rectal carcinomas [11-13].

Ovulation induction and oocyte retrieval can be successfully performed on transposed ovaries [14]. Studies on OT have rendered rather conflicting results, particularly those including patients who never went on to have radiotherapy. Nonetheless, retained ovaries carry a risk for symptomatic ovarian cysts and metastases to the ovaries from the primary site [15]. Yet, significant uncertainty exists regarding the efficacy of this procedure from observational studies with small sample sizes, which makes it difficult to counsel women accordingly. To ascertain the efficacy of OT, we conducted a systematic review and meta-analysis of the proportion of women who underwent OT with ovarian function preservation, symptomatic or asymptomatic ovarian cysts and metastatic ovarian malignancy.

## Materials and methods

### Study Identification

The population of interest in this review included premenopausal women with a diagnosis of a gynaecological malignancy who might require radiotherapy in addition to surgery. Patients who had OT without the need for adjuvant radiotherapy and who underwent unilateral OT were also included.

The intervention was OT and the outcomes included ovarian function preservation, metastatic ovarian malignancy and symptomatic or asymptomatic ovarian cysts. MEDLINE, EMBASE and The Cochrane Library were searched for articles published between January 1980 and April 2014. We used the following search strategy combining text and Medical Subject and Emtree Headings terms: women OR female AND ovar* transposition’ OR oophoropexy AND ovarian preservation OR ovarian function OR premature ovarian failure OR ovarian cysts OR metastases. No language restrictions were applied. Studies of women who had gynaecological surgery for benign indications resulting in ovarian cessation were not included in the review. References of selected studies were searched for articles not identified by the electronic searches, in an attempt to find additional citations. Review articles were not screened for additional citations. Case reports or series with fewer than 3 cases were excluded.

### Study selection and data extraction

The following information were extracted; publication date, type of study, duration of follow up, type of OT, retention of ovarian function, incidence of metastasis, ovarian cysts formation and associated complications. Studies were selected in a 2-stage process. Firstly, titles and abstracts from the electronic searches were screened by 2 independent reviewers (KG and TI) and full manuscripts of all citations that met the predefined selection criteria were obtained. Subsequently these articles were evaluated in full text for each of the 3 objectives to make the final selection. In case of duplicates, the most recent or complete publication was selected. Any disagreements were resolved by consensus or arbitration by a third reviewer (AL). Summary (aggregate) outcome data extracted from each study were created in 2×2 tables (AL). Preservation of ovarian function was assessed by patient's symptoms and serum FSH, LH, E2 levels. Only patients for which follow up was available were included in the meta-analysis. The Methodological Index for Non-Randomized Studies (MINORS), which assesses the quality of the included studies, was implemented [16]. For reporting of results, we followed the recommendations of Meta-analysis of Observational Studies in Epidemiology (MOOSE) [17].

### Statistical analysis

For the analysis of outcomes, we calculated the proportions of women who had their ovarian function preserved, had no ovarian cysts and no metastases in the transposed ovaries per total number of women undergoing OT. We computed the logarithm of the ratio and its corresponding standard error for each of the studies. A meta-analysis with inverse-variance weighting was performed using a random effects model. Forest plots were created for each outcome showing individual study proportions with confidence intervals (CIs) and the overall pool estimate. Heterogeneity was statistically evaluated using the I^2^ test. For funnel-plot asymmetry, we performed the Egger’s weighted regression test. Statistical analyses were performed using Stata 12.0 (Stata Corp, College Station, TX).

## Results

**Figure 1:**
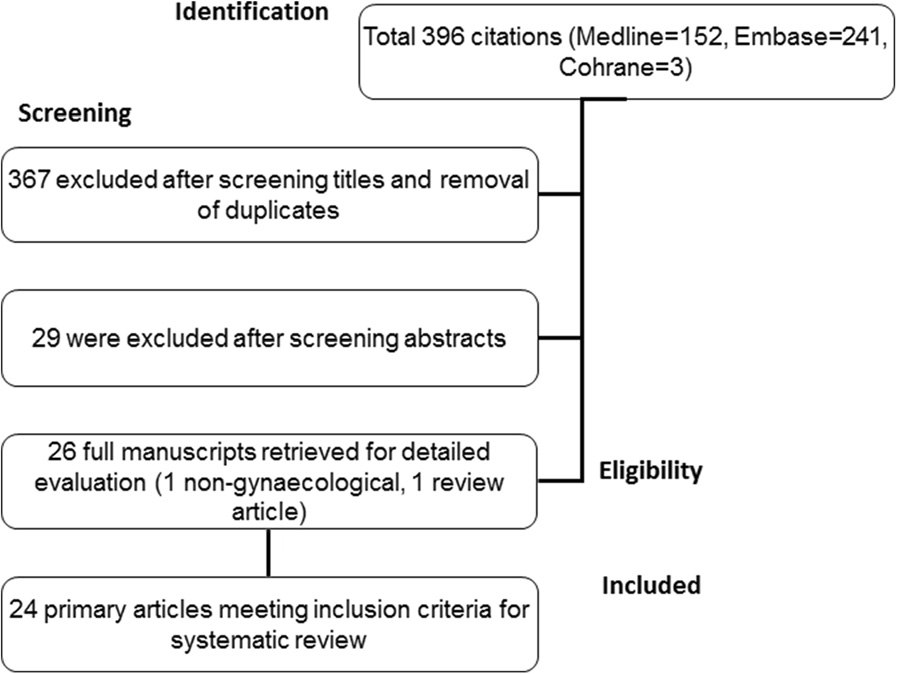
Study selection process.

**Figure 2:**
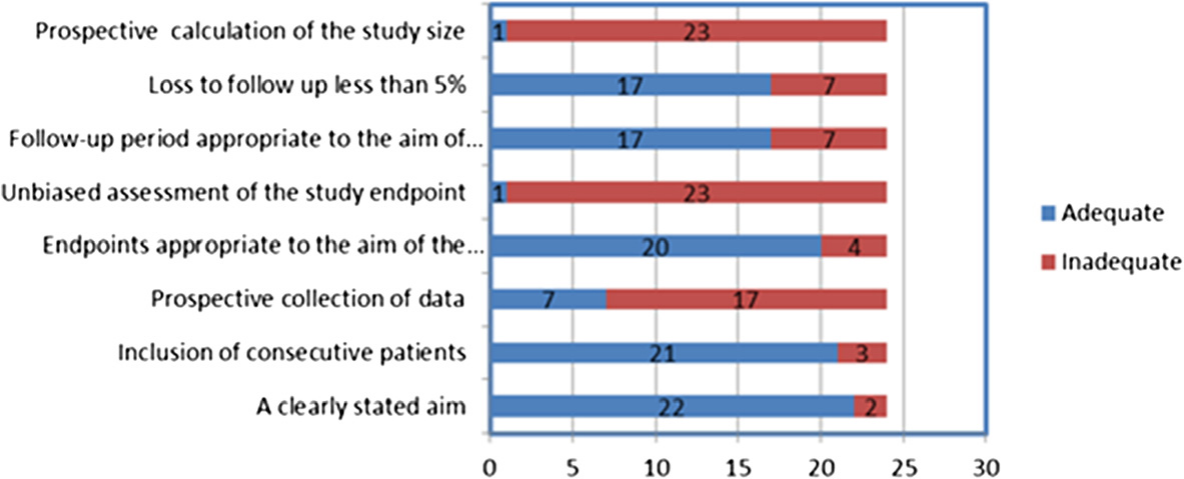
Quality assessment of the observational studies (MINORS criteria).

The electronic search strategy initially yielded 396 citations. The selection process for included articles is shown in Figure 1. We retrieved 26 articles for full text examination and we further excluded a review article [18] and an article with unrelated population as it was not referring to gynaecological malignancy [19]. As a result, 24 primary studies, reporting on 892 women who underwent OT were included in this review. The main study characteristics are shown in Table 1. The quality assessment of the studies in the MINORS checklist is shown in Figure 2. All studies were observational. In brief, 7/24 (29.1%) of studies were prospective including consecutive patients in 21/24 (87.5%). There was an adequate definition of outcomes in 20/24 (83.3%) studies. No studies had a blinded assessment of the outcomes or performed a prospective calculation of sample size. The mean follow-up was longer than 12 months in 19/24 (79.1%) of studies.

**Table 1:** Characteristics of studies

**First author and year of publication**	**Study design**	**Population**	**Intervention**	**Outcome(s)**	**Follow up (median, range)**
**Hodel 1982 **[7]	Retrospective	Women with vaginal (n = 2) and cervical (n = 7) cancers	Open-lateral OT in patients undergoing radical surgery followed by pelvic irradiation (n = 9)	Ovarian function by clinical symptoms and FSH levels	NR
**Husseinzadeh 1984 **[20]	Prospective	Women with cervical (n = 39) and vaginal (n = 1) cancers	Open–lateral OT in patients with RH + lymphadenectomy (n = 22), primary radiotherapy alone (n = 14) and radiotherapy following surgery (n = 4)	Ovarian function by FSH, LH levels, 15/22 from surgery only group were included for which FSH levels were available	NR
**Ploch 1988 **[21]	Prospective	Women with cervical cancer (n = 22)	RH with OT of one or both ovaries outside the pelvis (n = 5) followed by adjuvant radiotherapy (n = 17), BR only (n = 5) and BR _+_ teletherapy (n = 12)	Ovarian function by FSH, LH, E2 and progesterone levels, additional analysis of location of transposed ovaries	13 (2–23)
**Owens 1989 **[22]	Retrospective	All but 3 patients had early stage cervical cancer (n = 14)	All but one had bilateral open OT to the paracolic gutters (n = 14) in addition to RH, post-operative radiotherapy (n = 8)	Oestrogen deficiency symptoms, metastatic disease or required reoperation secondary to new ovarian pathology.	18
**Chambers 1990 **[23]	Retrospective	Premenopausal women with cervical cancer stage IA and IB (n = 84)	Lateral OT in addition to RH (n = 25) compared to non-OT group (n = 59)	Symptomatic ovarian cysts and symptoms of menopause by FSH and LH levels	14 (2–23)
**Van Beurden 1990 **[24]	Retrospective	Women with cervical cancer (n = 44)	Open intraperitoneal OT in a lateral and cranial direction (n = 44): In 16/44 women, only one ovary could be preserved and transposed and radiotherapy (n = 6)	Menopausal symptoms, measurement of FSH where available (n = 6)	23 (10–36)
**Chambers 1991 **[25]	Retrospective	Women with stage 1 cervical cancer (n = 38)	Open (sc) lateral OT (n = 38) as part of their initial operative procedure and post-operative radiotherapy (n = 14)	Ovarian function by FSH/LH, ovarian preservation directly related to estimated scattered dose to ovaries, symptomatic ovarian cysts by USS	35
**Anderson 1993 **[26]	Retrospective	Premenopausal women with early stage cervical cancer (n = 104)	Open-lateral OT (n = 82) , post-operative radiotherapy (n = 24), comparison with non-OT group (n = 22)	Retention of ovarian function, symptomatic ovarian cysts and metastases	44
**Bidzinski 1993 **[27]	Retrospective	Women with stage Ia and Ib carcinoma of the cervix (n = 48)	RH with OT (n = 48), EBRT (n = 15) and BR (n = 24)	Effect on ovarian function	40 (10–72)
**Feeney 1995 **[28]	Retrospective	Women with stage I-IIa cervical cancer (n = 132)	Lateral OT at the time of RH (n = 132), post-operative radiotherapy (n = 28)	Menopausal symptoms, FSH levels and adnexal pathology, Ovarian function is reserved only in 50% of patients with post-operative BR	24
**Clough 1996 **[9]	Prospective	Women with cervical cancer (n = 17)	Laparoscopic unilateral OT (n = 17) post-operative BR (n = 14 ) and EBRT + BR (n = 3)	Evaluation of ovarian function by clinical and laboratory criteria, 100% ovarian preservation in patients younger than 40 years old	23 (12–33)
**Covens 1996 **[29]	Retrospective	Patients with 1B cervical cancer prior to radiation therapy (n = 3)	Laparoscopic OT (n = 3) and had intarcavitary radiation desiring preservation of fertility.	Menstruating regularly after completion of treatment with serum FSH in the normal premenopausal range.	32
**Fujiwara 1997 **[8]	Retrospective	Description of a new technique for OT (n = 27), women with cervical cancer only were included (n = 12)	Open (sc) OT ovary (benign = 15, cancer = 12) and post-operative EBRT (n = 10) and BR (n = 1)	Cyst formation, symptoms of menopause with FSH levels measurement	26 (10–44)
**Morice 1998a **[30]	Retrospective	Women with 27 vaginal cancers, 9 ovarian dysgerminomas and 1 pelvic sarcoma	Laparoscopic OT	Ovarian function, cysts and prognosis for fertility	6
**Morice 1998b **[31]	Prospective	Only 14/ 24 were included as they were repeated in other paper published by the same author and 4 non gyanecological malignancies, 12 clear cell vaginal and cervical cancers, 1 vaginal adenocarcinoma, 1 dysgerminoma	Laparoscopic OT (n = 14), BR (n = 13) and EBRT (n = 5)	Clinical and laboratory follow-up tests of ovarian function and clinical pregnancies.	6
**Morice 2000 **[32]	Prospective	Women with cervical cancer (n = 107)	Laparoscopic bilateral OT to the paracolic gutters with RH and lymphadenectomy only (n = 11), with 60 Gy of vaginal BR along with surgery (n = 59) or surgery, BR and 45 Gy of EBRT (n = 25)	Ovarian function: by clinical symptoms, FSH, E2 level, 12 patients were lost to follow up, ovarian cysts: by USS	31 (10–56)
**Buekers 2001 **[33]	Retrospective	Women with cervical cancer (n = 80)	Open OT to one or both ovaries at the time of exploration for RH or staging lymphadenectomy, postoperative irradiation (n = 26)	Ovarian function by FSH, report of cyclic signs and menopausal symptoms, analysis of estrogen effect to vaginal epithelium	85 (43–126)
**Olejek 2001 **[34]	Retrospective	Women with cervical cancer for which follow up was available (n = 44)	Open OT, comparison of ovarian preservation between RT and non-RT groups	Ovarian function by FSH, LH, E2, PRL, testosterone, ovarian cysts by USS	60
**Yamamoto 2001 **[10]	Prospective	Women with cervical cancer (n = 56) Regression analysis of risk factors for ovarian metastases	Open OT during RH only (n = 30), with pelvic irradiation (n = 26)	Ovarian function by basal bosy temperature, FSH, E2 and PG, regression analysis of risk factors for ovarian metastases	12
**Nagao 2006 **[35]	Retrospective	Comparison between OT (n = 27)and 2 non-OT groups (n = 59) for ovarian preservation	Open OT following RH (n = 27)	Ovarian function by FSH	65
**Pahisa 2008 **[36]	Prospective	Women with 1b1 cervical cancer (n = 28)	Laparoscopic OT with no RT(n = 16), BR (n = 7) and EBRT + BR (n = 5)	Ovarian function by clinical symptoms and FSH and E3; follow up available for 24/28 patients, ovarian cysts by annual surveillance abdominal CT	44
**Al-Badawi 2010 **[37]	Retrospective	Women with cervical cancer (n = 15)	Bilateral laparoscopic OT to the paracolic gutters with uterine preservation followed by pelvic irradiation (n = 15)	Ovarian function by clinical symptoms and FSH	33
**Han 2011 **[38]	Retrospective	Women with cervical cancer (n = 29), comparison with non-OT group	OT in cervical cancer patients (n = 29) prior to pelvic irradiation	Ovarian function by E2 and FSH, 19/29 patients were included for which hormonal levels were available	17.2
**Hwang 2012 **[39]	Retrospective	Women with cervical cancer (n = 53), 39/53 patients were included	Open (n = 19) and laparoscopic (n = 34) OT to the paracolic gutters with primary chemoradiotherapy only (n = 3), with RH and lymphadenectomy (n = 33) followed by adjuvant RT (n = 23), with lymphadenectomy followed by primary chemoradiotherapy (n = 17)	Ovarian function by clinical symptoms and FSH,14/53 patients were lost on follow up or FSH not available	39.8

sc = Subcutaneous.

NR = Not reported.

OT = Ovarian transposition.

In the majority of the studies, the procedure was performed in patients less than 40 years of age. Surgery was by laparotomy or laparoscopy. In 2 studies the ovaries were transposed to the subcutaneous tissue [8,23]. The vast majority of patients (n = 828) had cervical cancer; 43 patients had vaginal cancer, 10 patients had ovarian dysgerminomas and 7 patients had pelvic sarcomas. There were 4 cases of pelvic lymphomas.

Women included in the analysis had a diagnosis of cancer and their ovaries transposed. A total of 428 women had surgery alone in the form of radical hysterectomy (RH) ± pelvic lymphadenectomy (PLND) ± upper vaginectomy ± paraaortic lymph node dissection (PALND) (Group A). 143 had post-operative brachytherapy (BR) (Group B). 321 had post-operative external beam radiotherapy (EBRT) ± BR (Group C). The follow up ranged from 2 to 126 months (Table 1).

### Preserved ovarian function

**Figure 3:**
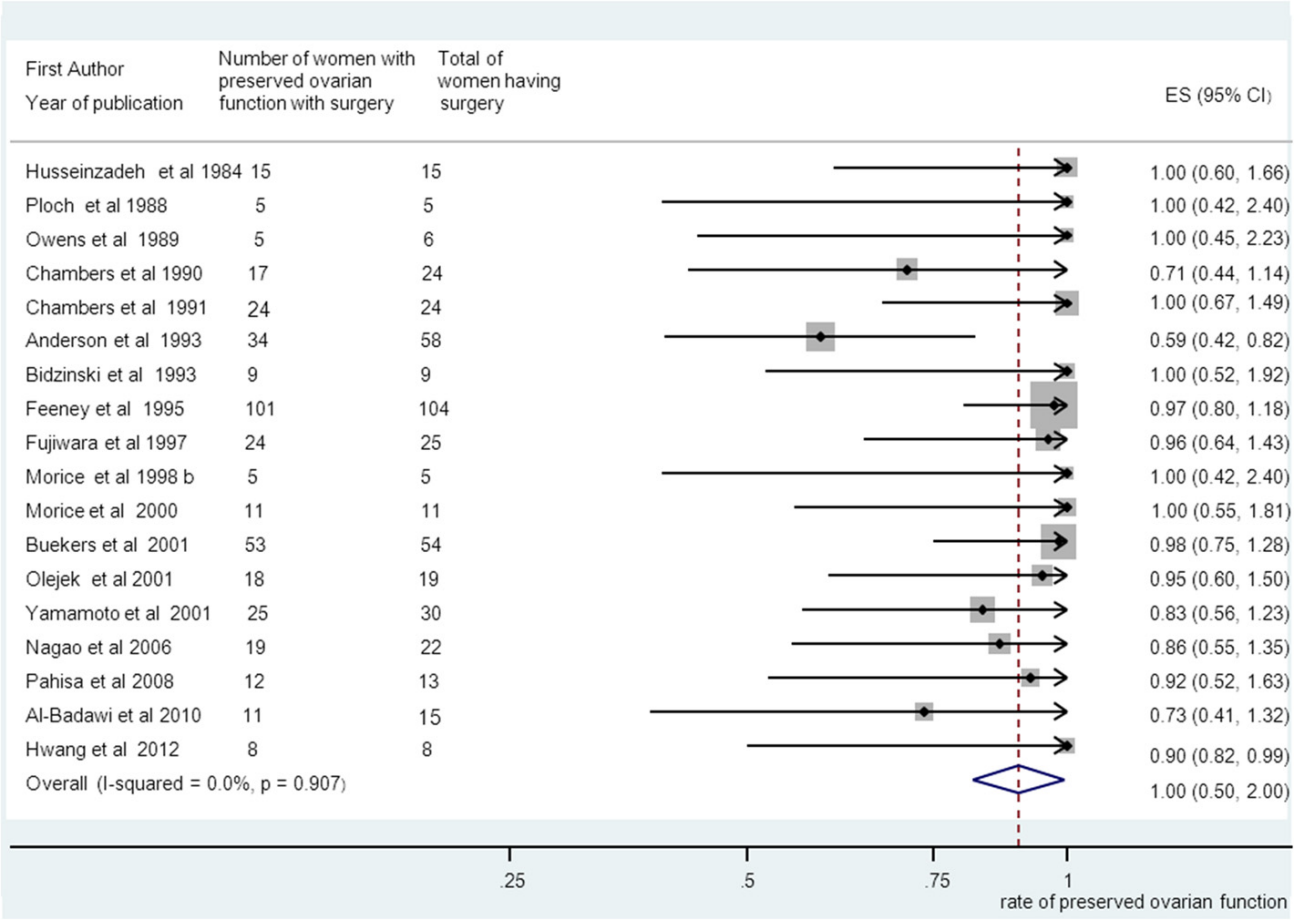
**Ovarian preservation and surgery only group.** Forest plot showing the proportions of women (with confidence intervals) with preserved ovarian function following ovarian transposition who had surgery alone.

**Figure 4:**
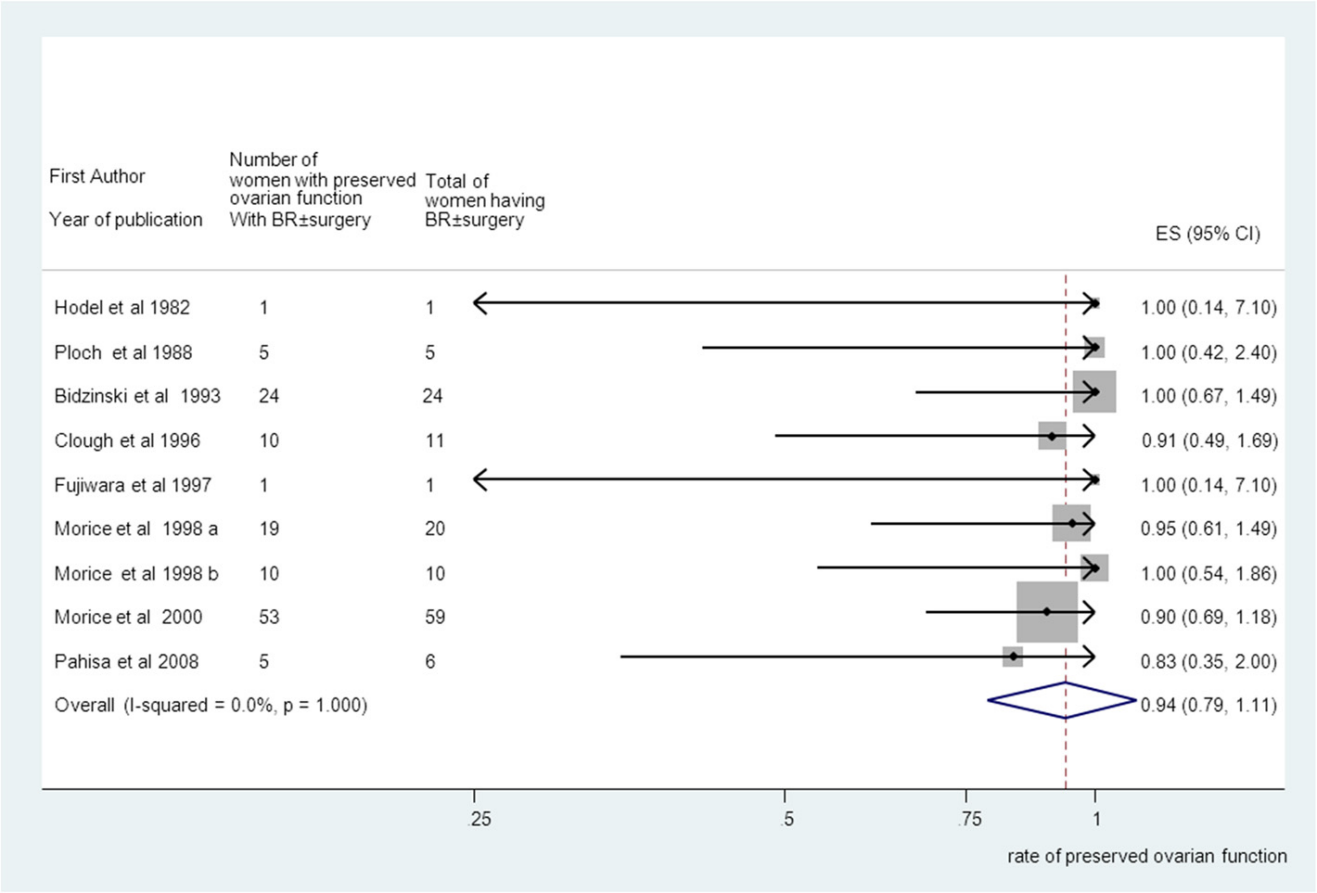
**Ovarian preservation and brachytherapy (BR) ± surgery group.** Forest plot showing the proportions of women (with confidence intervals) with preserved ovarian function following ovarian transposition who had brachytherapy (BR) ± surgery.

**Figure 5:**
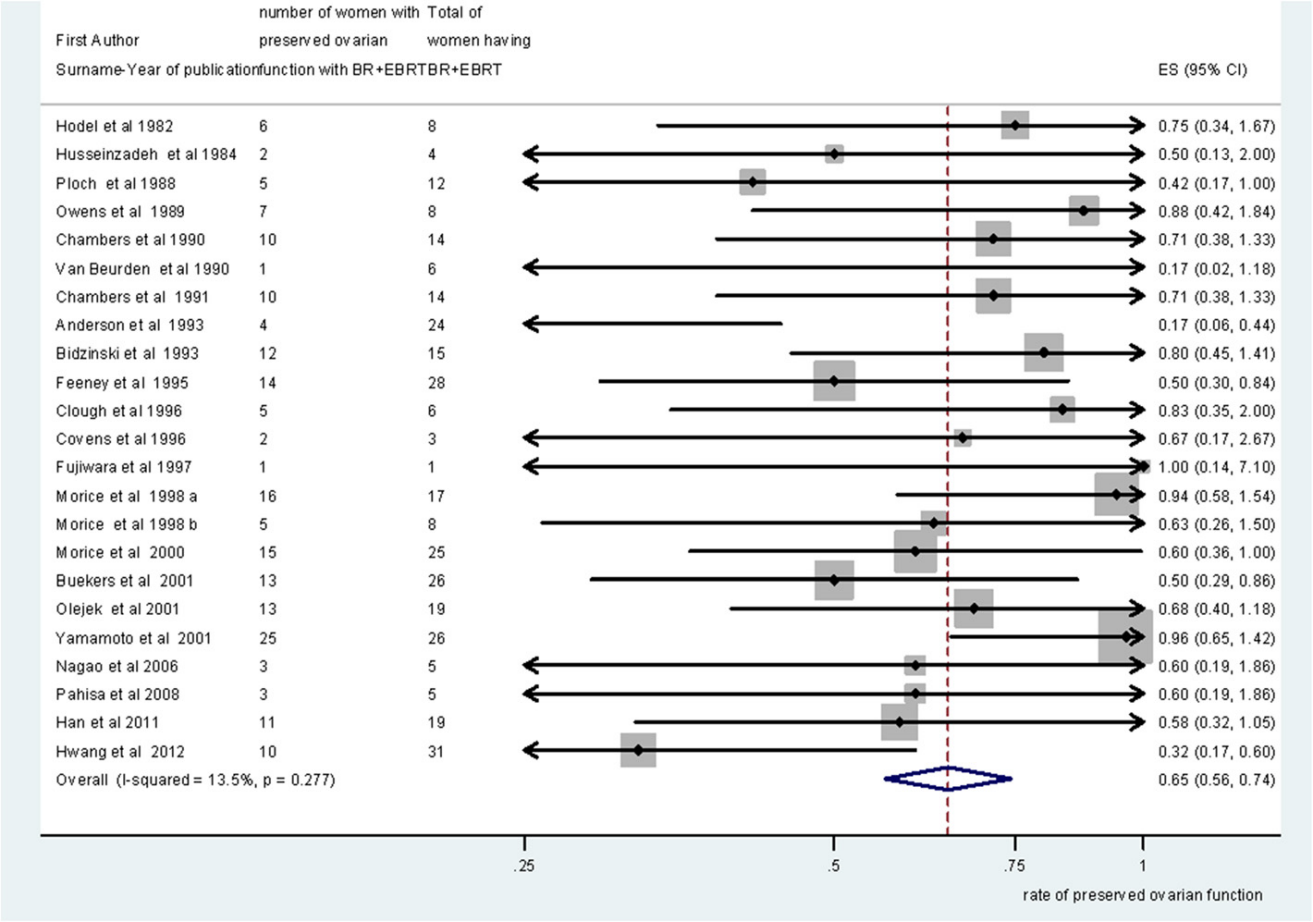
**Ovarian preservation and external beam radiotherapy (EBRT) + surgery ± brachytherapy (BR) group.** Forest plot showing the proportions of women (with 95% Confidence Intervals) with preserved ovarian function following ovarian transposition who had external beam radiotherapy (EBRT) + surgery ± brachytherapy (BR).

**Figure 6:**
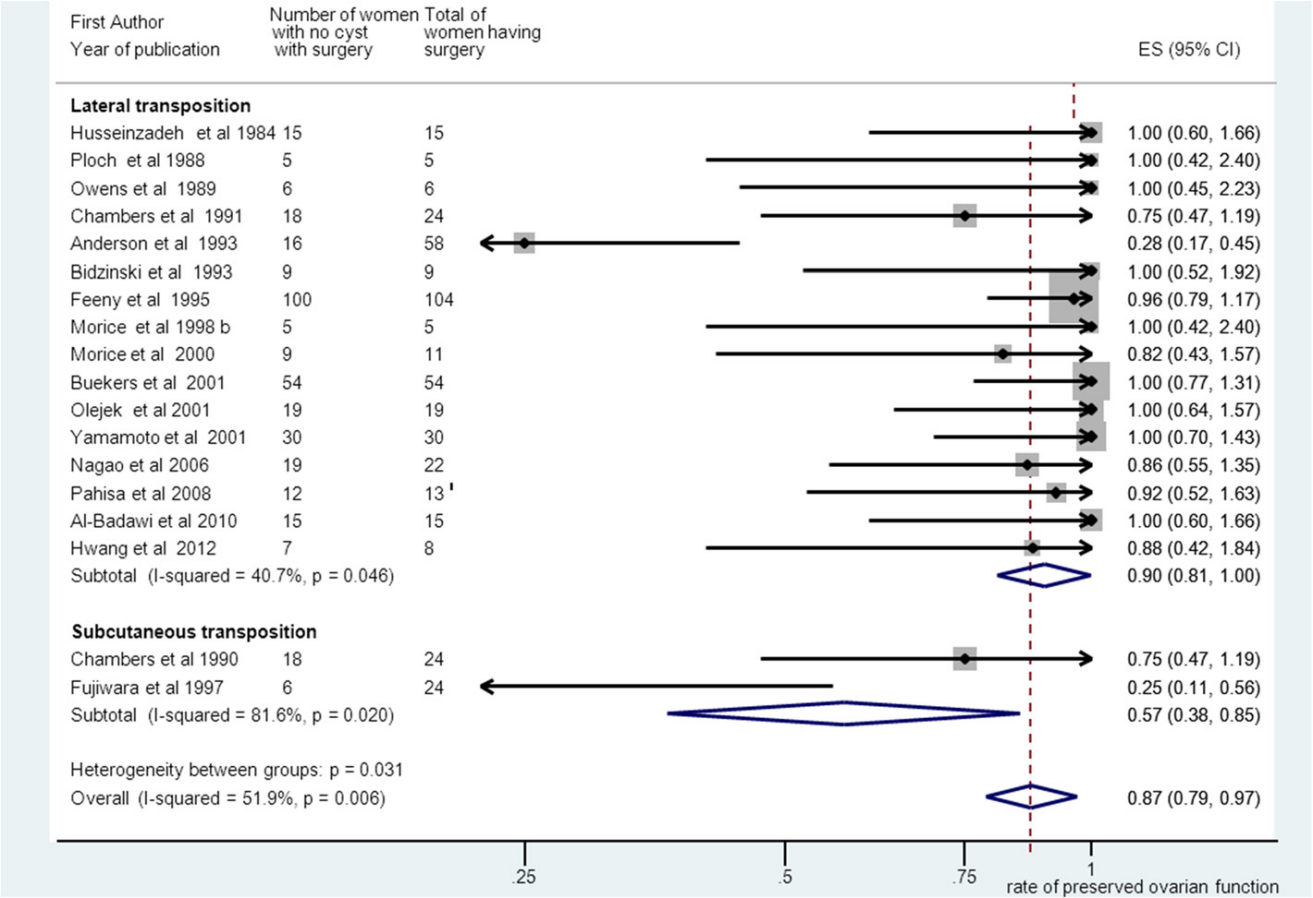
**Funnel plot of the random-effect estimates of the individual studies for ovarian preservation.** The vertical line indicates the random-effects summary estimate (using inverse-variance weighting) while the sloping lines indicate the 95% confidence intervals. Little heterogeneity was observed.

Pooling of results from 18 studies (n = 428 women) that reported ovarian function as an outcome in group A rendered a summary proportion of 90% (95% CI 82–99) for ovarian function preservation with no significant variation across the studies (I^2^ = 0.0%, p = 0.9) (Figure 3). The summary proportion from 9 studies (n = 143 women) for ovarian function preservation was also 90% (95% CI 79–111) for group B with no statistical variation across the studies (I^2^ = 0.0%, p = 1) (Figure 4). Pooling of results from 23 studies (n = 321 women) in group C rendered a summary proportion of 65% (95% CI 56–74) for ovarian function preservation with no significant variation across the studies (I^2^ = 13.5, p = 0.27) (Figure 5). For the outcome of ovarian function preservation no statistical significance for small study effects and plot asymmetry was found (Figure 6).

### Non metastases to the transposed ovaries

Pooling of results from 18 studies in group A, 10 studies in group B and 23 studies in group C reported no metastases to the transposed ovaries (95% CIs 90–111, 85–118 and 90–112 for groups A, B and C respectively). No significant variation across the studies for the 3 groups was observed (I^2^ = 0.0%, p = 1). In absolute numbers, only 1 study reported 2 recurrences in group A. These 2 patients had adenoid cystic carcinoma and cervical adenocarcinoma respectively. They underwent surgery for recurrent cancer 19 and 27 months after primary RH and both had disseminated disease involving transposed ovaries.

### No ovarian cyst formation

**Figure 7:**
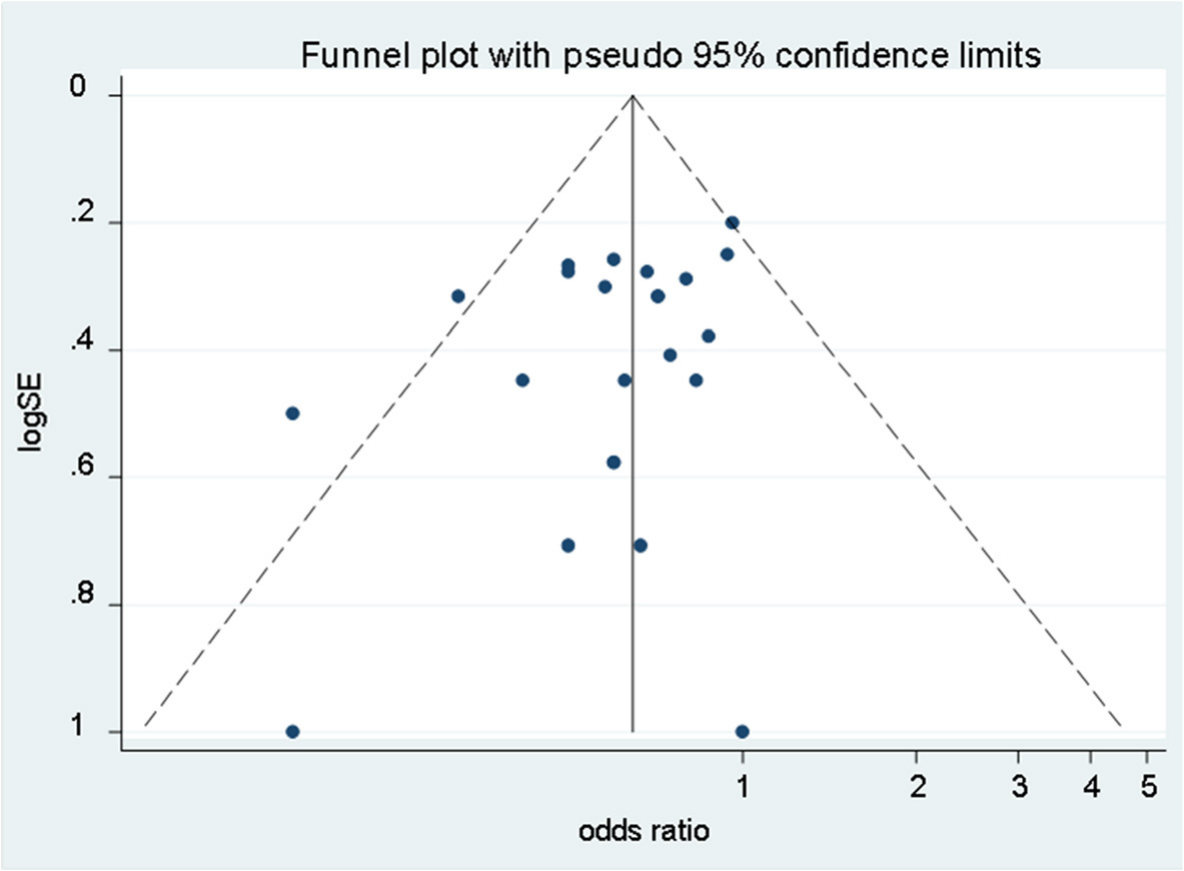
**Subgroup meta-analysis for no ovarian cyst formation and surgery group alone.** Forest plot showing the proportions of women (with 95% Confidence Intervals) with no ovarian cysts between subgroups of those with lateral ovarian transposition versus subcutaneous ovarian transposition. All patients had surgery alone.

Pooling of results from 18 studies that reported no ovarian cyst formation as an outcome in group A rendered a summary proportion of 87% for no ovarian cyst formation (95% CI 79–97). There was significant variation across the studies (I^2^ = 51.9%, p = 0.006). Interestingly, subgroup meta-analysis revealed that women undergoing subcutaneous OT (n = 2 studies, weight = 6.61%) had a 57% rate of no cyst formation compared to 90% on those (n = 16 studies, weight = 93.4%) undergoing “traditional” lateral OT (95% CI 38–85) versus (95% CI 81–100). However, variation across the studies for both subgroups was significant (I^2^ = 51.9%, p = 0.006) versus (I^2^ = 40.7%, p = 0.046) (Figure 7).

The summary proportion for no ovarian cyst formation in group B (n = 10 studies) was 84% (95% CI 70–101), (I^2^ = 0.0%, p = 0.885). No ovarian cyst formation was observed in the subgroup of patients with subcutaneous OT (n = 1 study, weight = 0.85%) (95% CI 70–110). Pooling of results from 23 studies in group C rendered a summary proportion of 95% for no ovarian cyst formation (95% CI 85–106) with no significant variation across the studies (I^2^ = 0.0%, p = 1). Subgroup meta-analysis between patients undergoing subcutaneous versus “traditional” OT revealed no difference in ovarian cyst formation rate ((95%CI 55–158) versus (95% CI 84–106)). No significant heterogeneity was observed across the studies for both subgroups.

## Discussion

The main aim of OT is to maintain ovarian function in premenopausal women treated with RT. It is now established as a simple and reliable method with reduced morbidity [9]. Published data vary with regards to functional outcomes such as ovarian failure, ovarian cysts and metastases to the transposed ovaries. Our systematic review of 24 studies confirms and generalizes the concept that OT is associated with a high preservation of ovarian function, an acceptable rate of ovarian cysts and a low risk of metastases in the transposed ovaries. To our knowledge, this is the first systematic review to provide an overview of the efficacy of OT on the aforementioned outcomes.

Selection of patients with early cervical and other gynaecological cancers who would benefit from OT is challenging, as difficulty arises to decide which patients would require postoperative RT prior to the surgical procedure [40]. To overcome this problem, we examined 3 treatment groups: Group A consisted of those patients who had surgery only. Patients in group B had post-operative BR. Group C consisted of patients who had primarily EBRT following surgery ± BR. OT was the fixed variable for all groups. In the absence of control groups for each treatment modality, the approach allowed for an indirect comparison amongst surgery, BR and EBRT without the risk of increasing missing data.Following a comprehensive search strategy, we employed the MINORS criteria, as indicated for quality assessment of the included observational non-comparative studies. We took into account the specific weight of the studies and after checking for heterogeneity, we employed a random-effects model to combine the data across studies to control variability. However, the majority of studies were of retrospective design. They were all observational; hence most were not designed for the specific outcomes examined with the exception of ovarian function. There was little evidence of publication bias as shown by the funnel-plot symmetry (Figure 6). The primary studies did not stratify their results by confounding factors such as age and follow up and hence, adjustment for these factors was not possible. Therefore, a certain degree of clinical heterogeneity should be expected.

In our studies, ovarian function was assessed by patient’s symptoms and serum FSH levels. Ovarian dose tolerance depends on volume irradiated, total radiation dose and the fractionation schedule in addition to patient’s age at the time of treatment. A fractionated dose exceeding 24 Gy to the ovaries can produce permanent ovarian ablation [41]. We demonstrated that ovarian function was highly preserved in those patients who had no adjuvant RT irrespective of the type of transposition or the position of transposed ovaries [25]. Only 10% of patients in that group became menopausal whilst theoretically they should be 5% or less [42]. Notably, those patients who had BR ± surgery (group B) performed as good as the surgery only group (group A). However, the strength of this inference may be reduced by the wider confidence intervals in this group due to smaller numbers of patients. While, the proportions of patients who had BR with intact uterus in group B is not known, we speculate a “timing effect” of the OT prior to irradiation as opposed to simultaneously at surgery. If OT takes place at the time of an extensive surgery, the risk for vascular compromise to the ovary from trauma or RH should be higher. Yet, the incidence of ovarian failure appears to be related to the length of follow up with 7% failing within 3 years and up to 50% within 5 years [22]. We noted that mean follow up in group B was shorter compared to group A, which might be partly responsible for the performance of the BR group. Notably, we observed that ovarian function was better preserved in those patients who have BR only without EBRT, consistent with the study by Morice et al. [32]. A plausible speculation for this is that EBRT may be damaging the vascular supply to the ovary as it loops down the pelvic brim before ascending again out of the brim to the transposed position. Therefore, if transposing an ovary prior to EBRT great care should be made to transpose the pedicle in addition to the ovary.

Further comparison between OT and non-OT cancer groups is required to assess directly the efficacy of OT for ovarian function preservation. However, considering that older women appear more susceptible to permanent ovarian damage compared to young women, it is expected that the incidence of ovarian failure following anticancer treatment ranges between 30-50% [43]. Therefore, although a direct comparison was not possible in the absence of non-OT cancer groups, we report an apparent benefit from OT for all treatment groups.

We demonstrated that the risk of ovarian carcinoma affecting the transposed ovaries is extremely low. Several risk factors for ovarian involvement have been suggested [15]. It appears that non-squamous histology carries a higher risk than the squamous one. Sutton et al. reported an incidence of 0.5% in squamous cell carcinoma compared to 1.7% in adenocarcinoma [44]. Therefore, ovarian metastasis in early cervical cancer occurs very rarely [45]. Moreover, the incidence of port site metastasis is <1% [32], which would explain our results, as the majority of transpositions were performed laparoscopically.

Risk factors for cyst development include previous surgery, extensive ovarian mobilisation albeit the mechanism is unknown and gynaecological pathology such as endometriosis or pelvic inflammatory disease [23]. In that respect, It is not surprising that the incidence of ovarian cyst formation in the transposed ovaries was common in the surgery only group (13%). In the subgroup meta-analysis, the possible advantages of subcutaneous transposition such as early detection and easier diagnosis of ovarian cysts, easy surgical access to remove ovarian cysts and facilitation of in vitro fertilization [8] were outweighed by a trend towards higher rate of ovarian cyst formation (43%) possibly due to the more extensive ovarian mobilisation at the time of subcutaneous transposition [23]. The risk for cyst formation was comparable to the BR group. As expected, only 5% of patients in the EBRT group had ovarian cysts because of the well-established association between menopausal status and ovarian cyst formation. Symptomatic cysts were identified by imaging and were treated either conservatively or surgically. Surgical treatment included needle puncture, cystectomy or oophorectomy. Conservative treatment included analgesics, hormonal or expectant management.

In conclusion, we confirm the efficacy of OT in patients undergoing radio-surgical treatment of gynaecological malignancies with high preservation of ovarian function and negligible risk of metastases to the transposed ovaries despite rather common incidence of ovarian cysts. Surgery alone and post-operative BR groups performed best for the above outcomes. As quality of care remains an important issue in cancer care, careful expansion of patient selection could identify those premenopausal patients who would really benefit from this rather underutilised procedure.

## Competing interests

The authors declare that they have no competing interest.

## Authors’ contributions

KG participated in the study design, conducted the literature search, extracted the data and authored the manuscript. AL reviewed the extracted data, drafted the manuscript and participated in the data analysis. IG performed the data analysis and revised the manuscript. PP and KH critically appraised and revised the manuscript. TI conceptualized the study, participated in the primary analysis and revised the manuscript. All authors read and approved the final manuscript.
